# Systematic Review of Emotion Detection with Computer Vision and Deep Learning

**DOI:** 10.3390/s24113484

**Published:** 2024-05-28

**Authors:** Rafael Pereira, Carla Mendes, José Ribeiro, Roberto Ribeiro, Rolando Miragaia, Nuno Rodrigues, Nuno Costa, António Pereira

**Affiliations:** 1 Computer Science and Communications Research Centre, School of Technology and Management, Polytechnic of Leiria, 2411-901 Leiria, Portugal; rafael.m.pereira@ipleiria.pt (R.P.); carla.c.mendes@ipleiria.pt (C.M.); jose.ribeiro@ipleiria.pt (J.R.); roberto.ribeiro@ipleiria.pt (R.R.); rolando.miragaia@ipleiria.pt (R.M.); nunorod@ipleiria.pt (N.R.); nuno.costa@ipleiria.pt (N.C.); 2 INOV INESC Inovação, Institute of New Technologies, Leiria Office, 2411-901 Leiria, Portugal

**Keywords:** emotion recognition, computer vision, deep learning, systematic review, emotion detection

## Abstract

Emotion recognition has become increasingly important in the field of Deep Learning (DL) and computer vision due to its broad applicability by using human–computer interaction (HCI) in areas such as psychology, healthcare, and entertainment. In this paper, we conduct a systematic review of facial and pose emotion recognition using DL and computer vision, analyzing and evaluating 77 papers from different sources under Preferred Reporting Items for Systematic Reviews and Meta-Analyses (PRISMA) guidelines. Our review covers several topics, including the scope and purpose of the studies, the methods employed, and the used datasets. The scope of this work is to conduct a systematic review of facial and pose emotion recognition using DL methods and computer vision. The studies were categorized based on a proposed taxonomy that describes the type of expressions used for emotion detection, the testing environment, the currently relevant DL methods, and the datasets used. The taxonomy of methods in our review includes Convolutional Neural Network (CNN), Faster Region-based Convolutional Neural Network (R-CNN), Vision Transformer (ViT), and “Other NNs”, which are the most commonly used models in the analyzed studies, indicating their trendiness in the field. Hybrid and augmented models are not explicitly categorized within this taxonomy, but they are still important to the field. This review offers an understanding of state-of-the-art computer vision algorithms and datasets for emotion recognition through facial expressions and body poses, allowing researchers to understand its fundamental components and trends.

## 1. Introduction

The field of Artificial Intelligence (AI) has made significant strides in recent years, particularly in the areas of machine learning and DL [1]. Machine learning is a type of AI that allows machines to learn from data without being explicitly programmed, while DL is a subset of machine learning that uses artificial neural networks to model and solve complex problems [2,3,4].

One area where machine learning and DL have been applied is in the analysis of human emotions. The ability to communicate verbally and non-verbally through Natural Language Understanding (NLU) [5,6], facial expressions and body poses has become an increasingly popular topic in psychology and computer science [7,8]. With the advent of computer vision, machines are now able to analyze our emotions for several purposes, including accident prevention with drowsy driving detection, social robots, and obtaining customer feedback [9].

Visual human emotions can be divided into two main categories: facial expressions and body expressions [10]. Facial expressions themselves are subdivided into macro-expressions, which are the large, easily observable facial movements typically associated with emotions such as happiness, sadness, anger, and fear; and micro-expressions, which are rapid, involuntary facial expressions that often reveal a person’s true emotions despite their attempts to conceal them [11]. On the other hand, body expressions include static poses and gestures, which encompass the posture, orientation, and movements of the body below the neck [12,13].

However, expression recognition can be challenging in real-world scenarios, such as when attempting to recognize emotions from different facial/body angles. Traditional methods like the Facial Actions Coding System, which utilizes 46 Action Units (AU) corresponding to facial muscle movements [14]. Still, these methods typically require more effort to get better results, which makes invariant emotion recognition a challenge for conventional methods [15,16]. Despite these AUs being foundational in the development of emotion recognition systems, a review led by psychologist Lisa Feldman Barrett [17] in 2017 highlighted an issue: there is no reliably consistent method for predicting a person’s emotional state.

However, DL methods have revolutionized the way machines learn, significantly enhancing the efficiency of emotion recognition and improving machine communication [18,19]. These advancements suggest a shift towards more adaptable and robust techniques, which might help overcome the complexities and limitations inherent in traditional emotion detection methods.

In this review, we analyze relevant and non-group specific studies in the field of emotion recognition using DL methods in computer vision, based on a proposed taxonomy that describes the type of expressions used for emotion detection, testing environments, relevant DL methods, and datasets used. We divide expression types into macro and micro facial expression recognition and static pose and body gesture recognition.

While many studies propose using traditional emotion recognition methods, our systematic literature review focuses on DL methods, particularly in computer vision. DL methods such as CNNs are advantageous because they abstract from reality by searching for patterns in images, enabling them to reach the state of the art in object recognition (e.g., R-CNN), feature extraction (e.g., CNN for landmark detection), and classification (e.g., general and attentional CNNs) [20,21]. Attention mechanisms using ViT, region-based CNNs for object recognition, and CNNs, in general, are also discussed.

The results from the selected studies analysis will help researchers understand key concepts in emotion recognition using computer vision and DL methods, with great potential in several areas, including human–computer interaction, virtual and augmented reality, advanced driver assistance systems, and entertainment [9]. Many studies test different CNN architectures and identify combinations to improve the precision of current state-of-the-art DL methods. We also perform a literature review on DL methods using computer vision, considering several aspects such as the scope of expressions covered, test environments, main methods used, and classification of datasets used for non-verbal emotion recognition.

Using the PRISMA guidelines, we narrowed down our study selection from 522 studies to 77 specific studies. Most articles on emotion recognition refer to facial expressions, as the face is the primary actor in non-verbal communication [22].

### 1.1. Research Relevance

Emotion detection is a research area with use-cases in several fields such as healthcare [23], education [24], and entertainment [25]. Accurate detection of emotions can help diagnose and treat mental health disorders, facilitate personalized learning, and enhance user experience in gaming and virtual reality. Emotion detection can also assist in identifying customer satisfaction levels, detecting fraudulent behavior, and improving public safety by monitoring suspicious activities. Thus, the significance of emotion detection lies in its potential to transform various industries and improve the quality of life for individuals. However, despite its significance, there is a limited number of systematic reviews comparing newer DL methods, such as region-based and ViT methods, with commonly used techniques, such as CNN. To address this gap, this study provides a comprehensive guide that covers topics related to emotion recognition using CNN techniques. Researchers looking to explore these newer methods can benefit from this guide. Notably, the study covers both traditional and DL techniques for Facial Emotion Recognition (FER) and Pose Emotion Recognition (PER). Therefore, it is a valuable resource for advancing the field of emotion recognition and is recommended for anyone interested in this topic. As depicted in Table 1, most systematic reviews focus on FER using DL methods, such as CNN, Long Short Term Memory (LSTM), or traditional methods like Support Vector Machine (SVM). However, this study emphasizes the implementation of CNN, region-based, and ViT methods, for both FER and PER.

### 1.2. Research Questions

To conduct this review, we first carried out a preliminary study to define a group of what we consider to be the most important research questions (RQ) for emotion detection context using DL methods and computer vision, which was answer throughout this article.

- RQ1: What types of emotion expressions are addressed in the literature?
- RQ2: What are the deep learning methods utilized?
- RQ3: Which datasets are employed by relevant works?
- RQ4: Which performance improvement techniques can be employed in this context?

### 1.3. Contributions

Besides the conceptual value of the answers to the main research questions of this article, several other contributions are highlighted:

- The focus is on providing a general understanding of the state-of-the-art computer vision algorithms and datasets for emotion recognition and helping researchers understand the fundamental components and the trends in facial and body pose emotion recognition fields.
- We provide a brief review of several datasets that include images and videos for facial and body emotion recognition, along with their purposes and characteristics.
- The analyzed studies are categorized based on a proposed taxonomy that aims to describe the type of expressions used for emotion detection, the testing environment, the currently relevant DL methods, and the datasets used.
- Studies are classified by type of learning to easily understand what is used in each study examined.
- Problems of FER and PER are addressed to provide a broad overview of emotion recognition using computer vision.

### 1.4. Review Structure

In Section 2, the methods used in this systematic review are addressed. Then, Section 3, will describe the results obtained from the Systematic Literature Review (SLR), showing the results through relevant tables, as well as an overview of DL methods and datasets according to its research area. The research questions are discussed and analyzed in Section 4, and it also emphasizes the key takeaways. The relevant findings are presented in Section 5 along with recommendations for further research in the area.

## 2. Methods

**Figure 1:**
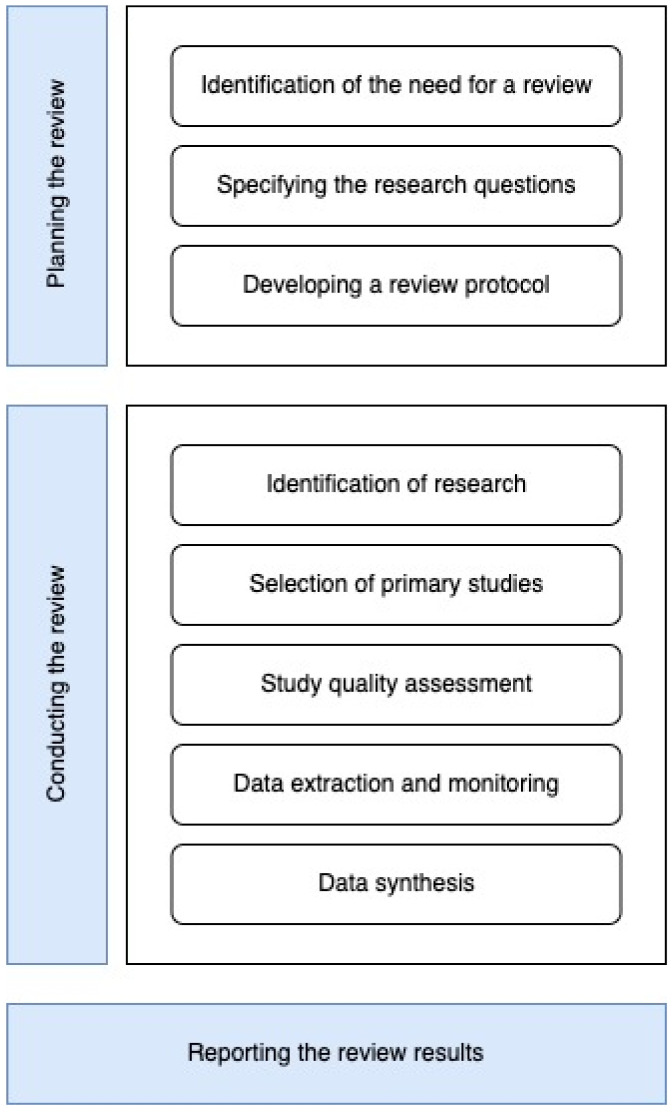
Systematic review process.

This study was conducted in three phases: Planning the review, conducting the review, and reporting the results of the review, as shown in Figure 1. In the first phase (planning the review), the need for writing this systematic review was defined, then the research questions, resulting in the review protocol. The second phase (conducting the review) was to perform the validation, identifying research questions that are answered during the SLR guiding and carrying out review stage. We then established a search strategy to find the initial research studies to review, followed by the primary selection of studies relevant to the research question. We then assess the quality of the studies to achieve better filtering. After selecting studies, the next step is data extraction. This involves collecting information from the most relevant studies. In this case, using a form to answer the original research questions. The results of the primary studies are then collected and summarized (data synthesis). Finally, the final results are reported (reporting the results of the review). In order to meet the objectives, the research team held weekly meetings.

### 2.1. Eligibility Criteria

The eligibility criteria considered for the study of the articles described in the Section 2.2 is governed by the following topics that are considered simultaneously.

Inclusion criteria:

- Empirical studies using facial or body pose emotion detection.
- Empirical studies using deep learning.
- Empirical studies using computer vision.
- Studies with paper, conference or articles formats.

Exclusion criteria:

- Studies using empirical verbal emotion detection.
- Studies using empirical deep learning in context other than emotion detection.
- Studies with empirical computer science context
- Studies with only abstracts.
- Studies not in English.

There is no time constraint on the selection of studies, as all DL methods can be relevant and contribute to this review. Thus, all those published before December 2023 and selected as described in Section 2.3 are taken into consideration.

### 2.2. Literature Search

To maximize the number of studies on this research topic from electrical databases of scientific documents, the search key includes several synonyms and a combination of the boolean expressions “AND” and “OR”. Consequently, the databases used were Scopus, IEEE Xplore, ACM Digital Library, and Web of Science. Regarding the composition of the search key, the expression refers to searching titles, abstracts, and keywords to find all papers dealing with emotion recognition from facial or body postures using DL methods through computer vision: “((pose OR body OR face OR facial) AND (emotion AND (recognition OR detection OR identification OR observation OR analysis OR extraction OR classification) AND (“Deep Learning” OR CNN OR CNNs OR ANN OR “Artificial Neural Network” OR Transformer) AND “Computer Vision”)”.

### 2.3. Study Selection

**Figure 2:**
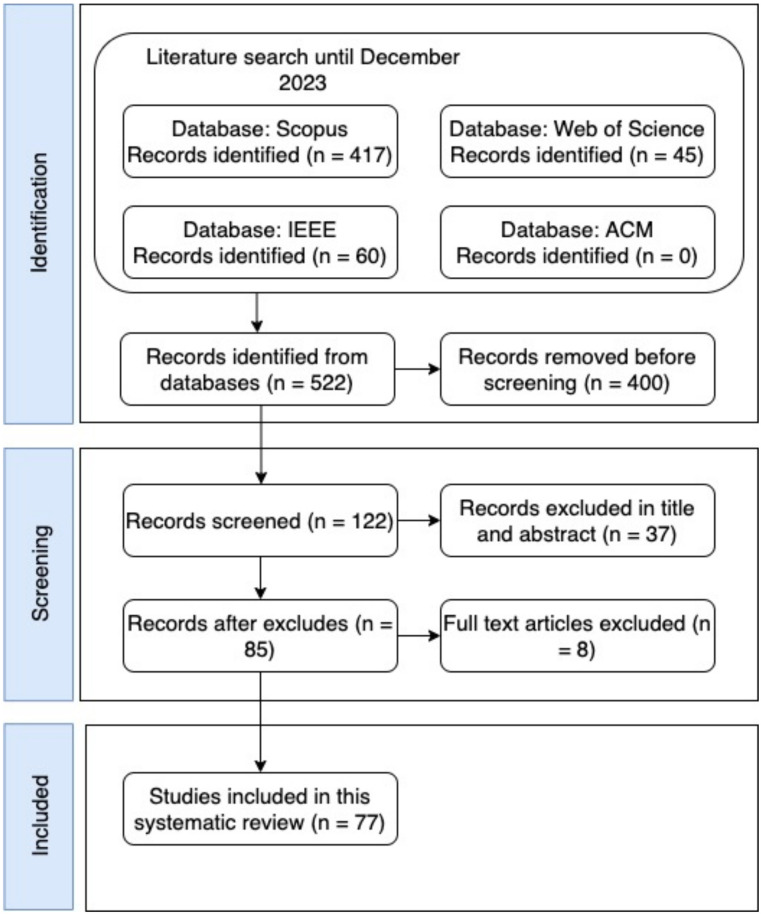
Study selection process.

This study selection was based on PRISMA [32] and the process for selecting primary studies is described in Figure 2. The search key consulted in Section 2.2 and used in four electronic databases (IEEE, Scopus, ACM, and Web of Science), resulted in 522 possible studies for analysis. Subsequently, 400 articles were excluded because they were duplicates or because they were not indexed, for a total of 122 for title and abstract analysis. From these 122 articles, 37 were removed because they were outside the scope of this review, leaving a total of 85 articles available for full-text quality assessment.

### 2.4. Study Quality Assessment

We conducted a quality assessment to select the most relevant studies on emotion recognition with DL using computer vision. Initially, we identified 85 potential documents for analysis and then carried out a quality assessment, with two of the most experienced team members answering nine questions based on the guidelines provided in [33,34]. Table 2 displays the quality assessment questions with the possible responses of 1 (yes), 0.5 (partially), and 0 (no) for each article.

We calculated the average of the nine responses for each study, and the results were categorized into high, medium, and low, as shown in Table 3. Out of the initial pool of documents, 77 studies received scores of medium or higher and were deemed relevant for review. The majority of studies were in the high, medium, and low categories, as expected from the filters applied earlier.

After much deliberation and meetings, only 8 studies were excluded, for receiving the lowest scores, leaving 77 studies for further analysis. In Section 3, we focus on studies rated as low or higher in Table 3, while only 14 studies received high or very high scores. This is mainly due to the specific research questions, where answers were often categorized as partial and studies rated as medium or low.

## 3. Results

**Figure 3:**
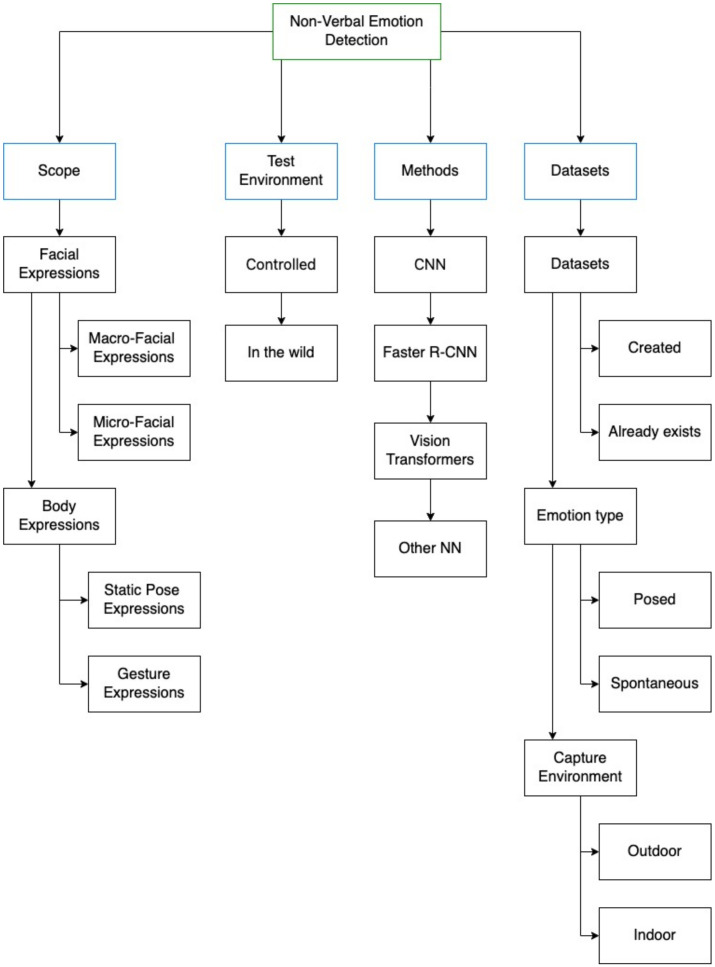
Results taxonomy.

This section presents a comprehensive summary of the findings from the literature review carried out on all the studies chosen in the preceding section. In order to thoroughly examine each study, we have devised a taxonomy, as depicted in Figure 3. This classification system takes into account various dimensions, drawing on the insights gleaned from the reviewed studies and the knowledge we have acquired throughout the process.

The first dimension to consider is the scope of the study, which includes the methods used to detect emotions, such as facial expressions or pose expressions. The second dimension relates to the test environment used in the study, which can be a real-world scenario (in the wild) [35] or a controlled environment, such as a researcher’s local computer. The third dimension pertains to the methodology and architecture adopted in the study, including the use of techniques such as CNNs, Faster R-CNN, or ViT. Finally, the last dimension concerns the datasets used, including whether they were created by the authors or by third party, for the studies created by the authors, as well as a classification of the type of emotions captured (natural/spontaneous or posed/forced), and the capture environment (outdoor or indoor).

According to psychological research, humans primarily judge other people’s communication behavior through the visual cues of their facial and body expressions; this is called non-verbal communication [36,37]. In this SLR, an expression refers to the movement that represents an emotion. Emotions can be concealed through a lack of movement, but can also be displayed through a forced or unintended expression [38]. As depicted in the scope dimension of Figure 3, this review aims to analyze four types of expressions: macro-expressions and micro-expressions from facial expressions and body expressions, divided into gestures and static pose expressions.

Visual emotional expressions can be categorized into facial and body expressions, each encompassing distinct types and mechanisms [39]. Facial expressions are further divided into macro-expressions and micro-expressions [38]. Macro-expressions are voluntary and encompass the six universal expressions: happiness, sadness, fear, surprise, anger, and disgust. As Shreve et al. [11] say, these expressions last from three-quarters of a second to two seconds and can manifest across multiple or single regions of the face, depending on the emotion. For example, surprise typically involves movement around the eyes, forehead, cheeks, and mouth, whereas sadness is more confined to the mouth and cheek areas.

Micro-expressions, on the other hand, are involuntary, subtle, and fleeting, usually within half of a second. These expressions might contain unique properties essential for detecting emotions in brief moments, making them potentially relevant for researchers in fields that require rapid emotion detection [40]. Micro-expressions can be classified into three types: simulated expressions, where there is no genuine expression; neutralized expressions, where a genuine expression is suppressed and the face remains neutral; and masked expressions, where a genuine expression is completely concealed by a falsified expression [38,41]. This paper will not discuss this type in the study revision.

Body expressions, categorized into static poses and gestures, offer additional layers of emotional insight. Gestures are dynamic and require analysis over time, often through sequences of frames, to capture the flow and transition of emotional states. Static poses, however, are captured in a single frame, representing a snapshot of an emotional state at a specific moment [39]. While research on static poses is less prevalent than that on facial expressions, they play a crucial role in scenarios where facial cues are limited or absent, such as in telephone conversations or situations where individuals’ faces are obscured or away from the camera [12,13]. Although these categories are not heavily researched, they might be important for addressing problems in emotion detection [30].

In addition to the scope of the study, the testing environment is also a dimension of the presented taxonomy. The studies aim to classify the environment of the proposed methods to understand whether they are designed for detecting emotions in a real-world context (in the wild) or a controlled context [35]. Typically, test results in controlled environments have much higher accuracy compared to tests conducted in uncontrolled environments (in the wild), making this task challenging [26].

Emotion recognition using DL methods typically involves a pipeline from data collection to the classification of new data. Collecting and annotating data is difficult and expensive, which hinders development. To overcome this problem, various strategies, such as transfer learning or fine-tuning, can be used [42], or even using Generative Adversarial Networks (GANs) as discussed by Shorten et al. [43]. Next, image preprocessing could be performed to improve data quality and robustness. This includes techniques like rotation and cropping for augmentation, as well as intensity normalization with filters to reduce image noise [44]. Reducing image noise improves the effectiveness of the feature extractor. This extraction is usually performed with neural networks that provide feature vectors and landmarks of key points on the face and body [20,45]. Based on the features extracted from the images, the classification process can now be performed, which requires a trained model. In this SLR, several classifiers are described, and the proposed models based on CNN architectures have shown the most potential [9].

DL methods continue to evolve, and recently, methods such as Faster R-CNN [46] and vision transformers [47] have appeared. The methods dimension aims to categorize and describe the approach used/developed in the study into CNN, R-CNN, and ViT as shown in Figure 3. These methods were the most used by the analyzed studies, and “Other Neural Network (NN)” methods are not considered in the categorization but are still explained in the text.

In recent years, the field of emotion recognition using computer vision and DL techniques has experienced a significant surge in development [46]. Previous research has evaluated methods for detecting emotions in images and videos, including older computer vision techniques such as R-CNN as well as newer techniques such as vision transformers and Faster R-CNNs [47]. These studies have shown that DL methods, especially those relying on CNNs and transformers, outperform traditional computer vision techniques in terms of accuracy and robustness. The method dimension is intended to categorize and describe the approaches developed in the study into CNN, Faster R-CNN, and ViT, as shown in Figure 3. Again, “Other NN” methods are not included in the categorization but are described textually.

CNNs are a type of DL algorithm commonly used in computer vision, including emotion recognition. CNNs are designed to automatically learn and extract features from images, which can then be used to make predictions about the emotional state of a person in an image [48,49]. In the context of emotion recognition, CNNs can be trained on large datasets of images and corresponding emotional labels to learn the patterns and features associated with different emotions. The trained CNN can then be used to make predictions about the emotions of new images [50].

The use of CNNs in emotion recognition has several advantages over traditional image processing methods. For example, CNNs can learn to automatically identify relevant features in images without requiring manual feature acquisition, which can be a time-consuming and error-prone process. In addition, CNNs can process large amounts of data and learn complex relationships between inputs and outputs, making them well suited to the task of recognizing emotions from images [48]. In addition, advancements in DL, particularly through the integration of techniques like CNNs and other complementary methods, allow for better variable pose handling, lighting conditions, and other environmental factors that can affect the appearance of images [51]. Overall, the use of CNNs has significantly improved the accuracy and speed of emotion recognition compared to traditional methods [52].

R-CNN is a computer vision algorithm for object detection. It consists of three main components: region-based proposal, feature extraction, and object classification. It is an older approach in DL and has since been improved by faster and more accurate algorithms such as Faster R-CNN and Single Shot MultiBox Detector (SSD) implemented in You Only Look Once (YOLO). Although R-CNN was originally developed for object recognition, it can also be applied to emotion recognition in images and videos by training it on a dataset of faces with emotions. The network identifies regions of interest in the image, such as a face [48,53].

ViT are a type of neural network architecture based on the transformer architecture originally introduced in the field of Natural Language Processing (NLP). In recent years, they have been adapted for computer vision tasks such as image classification, object recognition, and segmentation. In the context of emotion recognition, a vision transformer can be trained on a dataset of faces labeled with emotions, taking the entire image as input and producing a classification of the emotion shown in the image. Unlike older computer vision techniques such as R-CNN, vision transformers do not require predefined regions of interest and can use the global context in the image to make a prediction. This can lead to improved accuracy and robustness in emotion detection compared to other approaches [54,55].

Fine-tuning, hyper-parameter tuning, and batch normalization are optimization techniques that might improve deep learning methods. Fine-tuning allows pre-trained models to be adapted to specific datasets, enhancing their predictive capabilities for specific tasks [56]. It is necessary to continuously adjust hyper-parameters and train multiple models with different value combinations, then compare their performance to select the best one. This makes hyper-parameter optimization a good technique for DL model architecture and training processes [57]. Batch normalization normalizes layer inputs to accelerate training by reducing internal covariate shift, enabling higher learning rates, and acting as a regularizer while stabilizing the learning process [58].

The last dimension of the taxonomy represents the datasets used in the studies for the development of the methods. This dimension distinguishes between datasets captured by the study’s authors or obtained from third parties. If the datasets were built by the authors, an in-depth analysis is conducted on certain key characteristics. These include the type of emotion captured, whether posed/forced or spontaneous, with spontaneously collected data typically providing higher real-world accuracy than forced data [59]. Another crucial consideration is the environment in which the data were collected, specifically, whether it was an indoor or outdoor setting. These elements are crucial for the adaptability and generalization of DL methods, which are fundamental for their performance, and are largely influenced by the characteristics of the training dataset.

### 3.1. Common Datasets

In the context of emotion detection, diverse datasets serve as essential resources for researchers and practitioners to train and evaluate their models using DL methods. In this section, we will explore the most prominent datasets employed in emotion detection, such as the AffectNet dataset, FER-2013 dataset, CK+ dataset, and JAFFE dataset, among others, as detailed in Table 4. Each dataset exhibits distinct characteristics, varying in size, complexity, and annotation methodology, offering valuable assets for those aiming to develop and enhance emotion detection models. The objective is to provide a comprehensive overview of these key datasets, enabling readers to gain valuable insights into the current state of emotion detection research and make informed decisions when choosing appropriate resources for their projects.

Emotion detection research relies on diverse datasets, which are indispensable for training and evaluating models using deep learning methods. These datasets, detailed in Table 4, range in size, complexity, and annotation methodology, encompassing both macro and micro facial expressions, gesture expressions, and static pose expressions. They include images and videos acquired in controlled laboratory environments (posed) and dynamic real-world scenarios (in the wild), each offering a unique set of challenges and insights. The accuracy of facial emotion recognition is influenced by different factors such as social norms, cultural differences, and environmental conditions, highlighting the need for diverse and representative data. Micro-expressions, a subset of these datasets, are brief and subtle, requiring precise motion tracking for recognition. They often indicate hidden emotions and are critical in understanding human behavior. The datasets provide an extensive range of emotions, with some capturing maximum intensity emotions while others record a mix of emotional states, aiding in the advancement of emotion detection technologies.

### 3.2. Study Characteristics

This section summarizes the information collected during the analysis of each article selected in Section 2.4. The details are organized according to the dimensions of the taxonomy, and shown as abbreviations in Table 5. Based on those abbreviations, Table 6 provides a comprehensive summary of all the investigations analyzed, including the references of the articles, the scope of the problem addressed, whether or not the authors created a dataset, the architectures of the methods applied, the testing environments, and the publication years. Similarly, Table 7 shows abbreviations for Table 8 simplification which details the datasets proposed by the analyzed papers.

### 3.3. Literature Review

Human communication relies heavily on non-verbal expressions to grasp one another’s intentions. Generally speaking, individuals use their speech tones and facial expressions to infer other people’s emotional states, such as happiness, sorrow, and rage [9,138]. The difference between an emotion and an expression is that an emotion is something a person feels and an expression is something that is done to show emotion. There can be an emotion without expression occurring. But an expression is an indicator that an emotion is occurring [11,38]. The human body is full of expressions, from muscle movements of the face or the body itself. Facial expressions are divided into macro- and micro-expressions, this division is based on the expressiveness with which the movements are performed and the duration they occur. Body movements can be evaluated statically and using only one image, or the complete movement can be evaluated using a sequence of images corresponding to the movement [13,36]. In the following sections, we further address these types of expressions.

The detection of human emotions is a key process for the development of new technologies such as social robots; these have recently been a major focus of researchers, who face challenges such as the detection of emotions in the wild [26,139] because of variations in lighting, variations in expressions, and so on [66]. Thus, this section intends to describe the objective of each study in Table 6, and describe its methods, results, and conclusions dividing the studies by the emotion detection scope.

#### 3.3.1. Facial Macro-Expressions

Macro-expressions usually occur in several zones of the face and are easily observed. They last between three-quarters of a second and two seconds [11]. Therefore, it is usually easy to recognize these emotions. Depending on the expression, the distinctive features of a macro-level expression can be seen throughout the face [38]. This section describes approaches used by researchers to identify emotions from macro-expressions.

In the context of emotion detection, one understudied area is the real-time performance of proposed approaches, especially in terms of their effectiveness in real-world conditions like low light and varied head poses. Zaman et al. [61] propose a high Frames Per Second (FPS) method using an improved Faster R-CNN for Region of Interest (RoI) detection, an SVM for feature extraction, and transfer learning of NasNet-Large CNN pre-trained on ImageNet data with a custom-created dataset of driver emotions for classification. To evaluate the method, datasets were split using holdout cross-validation, with data randomly divided into a training set (70%) and a test set (30%), and also augmented. The method achieves accuracies of 98.48% on the JAFFE dataset, 99.73% on the CK+ dataset, 99.95% on the FER-2013 dataset, and 95.28% on the AffectNet. Addressing similar in the wild challenges, Yao et al. [89] introduce the Fine-Tuned Channel-Spatial Attention Transformer (FT-CSAT) model, which employs vision transformers with a structural backbone based on the CSWin Transformer, initially trained on the ImageNet-1K dataset. This backbone includes a channel-spatial attention module designed to enhance global feature extraction, addressing the CSWin Transformer’s limitation in handling global information due to its self-attention mechanism, which processes the input into smaller blocks. During fine-tuning, the model employs the Adam optimizer and the cross-entropy loss function. The FT-CSAT achieves accuracies of 87.58% and 88.05% on the Relaxed Affective Faces Database (RAF-DB) and FERPlus datasets, respectively.

A significant challenge in the field of emotion detection is the scarcity of datasets, particularly those derived from real-world scenarios. Addressing this gap, Li et al. [72] introduce the RAF-DB dataset for FER, which consists of 30,000 facial images from the wild. To create a base-line they propose the Deep Locality-Preserving (DLP)-CNN method, adding a supervised layer locality preserving loss to a scratch-built CNN to enhance feature discriminability, resulting in accuracies of 74.20% (DLP-CNN + Multiclass SVM) and 70.98% (DLP-CNN + Linear Discriminant Analysis (LDA)). Further contributing to this challenge, Márquez et al. [40] introduce the SL-Swin-T model, a compact version of the Swin Transformer. This model integrates Shifted Patch Tokenization (SPT) and Locality Self-Attention (LSA) techniques and was trained from scratch on small datasets, particularly CASME II and SAMM Long Videos, targeting both macro- and micro-expressions, achieving an overall F1-score of 0.1824 and 0.1357, respectively, on these datasets. The precision results were obtained through the Leave-One-Subject-Out (LOSO) cross-validation method, which is standard in expression recognition to handle small sample sizes effectively. The training configuration involved a standard batch size and learning rate optimized for small-scale datasets, with adjustments made during training to accommodate the specific challenges posed by the limited data size and diversity. Additionally, Lakhani et al. [83] address facial emotion detection in animations by creating a dataset from *Toy Story 1–4* and Pixar short films and employing a CNN with a noted accuracy of 77.65%. To mitigate potential overfitting, they used a holdout test set comprised of images from *Toy Story 4* and other Pixar shorts not included in the training set. To address data scarcity, Athanasiadis et al. [114] use Domain Adaptation Conditional Semi-Supervised Generative Adversarial Network (dacssGAN), a custom Generative Adversarial Networks (GAN), to improve audio-visual dataset classification. They use input and batch normalization, LeakyReLU activations, and Adagrad for optimization and L1 normalization in the loss function. In their experiments they used CREMA-D and RAVDESS datasets, achieving classification accuracies of 50.29% and 48.65%, outperforming the original datasets’ 49.34% and 46.90%.

Addressing the challenges posed by noise in emotion detection, Said et al. [102] develop a method for classifying macro-expressions in high-resolution images, employing a Face-Sensitive CNN (FS-CNN) that utilizes skin tone detection to remove backgrounds. The FS-CNN is trained on two datasets: CelebA, with 202,599 RGB images, and UMD faces, containing 367,888 images. The data are divided into 70% for training, 30% for testing, and 10% of the training data for validation purposes. The model achieves accuracies of 92.6% in face detection and 94.9% in emotion recognition respectively for the datasets. Furthering the advancements in feature extraction, Schoneveld et al. [45] present Deep Facial Expression Vector ExtractoR (DeepFEVER), a CNN-focused facial feature extractor, achieving 65.4% accuracy on the AffectNet dataset. The two-branched approach starts to be a recurring theme in recent studies. Mukhiddinov et al. [74] explore emotion detection in faces with masks, focusing on the upper part of the face, and introduce a two-branch CNN-based model for low-light image enhancement, achieving 69.3% accuracy on AffectNet. Similarly, Hayale et al. [86,140] implement a Siamese model with two branches using DenseNet121 in each, focusing on mapping images in feature space for improved emotion detection, and demonstrating accuracies up to 98.95% on CFEE-7 dataset. This approach highlights the effectiveness of bifurcated networks in handling complex facial recognition tasks in varied conditions. Continuing this trend, Zhang et al. [76] combine datasets Aff-Wild2, BP4D, and DFEW, utilizing a CNN for feature extraction and obtaining a 79.3% accuracy rate. Shabbir et al. [90] propose the Fine-Grained Bilinear CNN (FgbCNN) to enhance the generalization of facial features in expressions such as anger and fear, achieving high accuracies on multiple datasets.

The effectiveness of emotion detection systems heavily relies on the precision of feature extraction. In this context, Bellamkonda et al. [71] apply image processing techniques like Local Binary Pattern, Gabor Wavelets, and Local Directional Pattern for feature extraction and SVM for classification. This strategy achieves better precision results compared to Artificial Neural Network (ANN), with accuracies of 96.50%, 95.83%, and 93.83% on MMI, CK, and JAFFE datasets, respectively. To further enhance feature extraction and classification accuracy, Wu et al. [65] propose an Adaptive Feature Mapping built-in with a CNN, achieving accuracies of 89.84%, 96.24%, and 92.70% on CK+, RaFD, and ADFES. The model employs nine convolutional layers, multiple pooling layers, and three fully connected layers, with Local Response Normalization to optimize feature extraction.

Alagesan [66] emphasizes the effectiveness of Histogram-Oriented Gradient (HoG) and CNN for nuanced expression analysis. By integrating HoG descriptors with an adapted VGG-Face model, the research achieves impressive accuracies on the CK+, Yale-Face, and KFED datasets. Building on this approach, Fu et al. [92] introduce strategies with Memo Affinity Loss (MAL) and Mask Attention Fine Tuning (MAFT) for FER which were incorporated into an Architecture Attention ResNet (AAR) model that leverages channel and spatial attention fusion using CBAM modules, achieving 88.75% on RAF-DB, 65.17% on AffectNet-7, 60.72% on AffectNet-8. Meanwhile, Aparna et al. [125] propose a specialized framework for recognizing emotions in Indian classical dance, employing HoG for initial image processing, a CNN architecture derived from AlexNet for feature extraction, and SVM for the final classification, with a demonstrated accuracy on culturally specific datasets. Furthermore, Daniel et al. [118] conduct a comparative analysis of facial recognition technologies, including Faster R-CNN and SSD, along with emotion recognition methods like VGG-16, ResNet, and InceptionV3. They also used Rectified Adam (RAdam) and batch normalization to improve the performance of these deep learning models. The study uses a dataset comprising images from CK+, JAFFE, KDEF, and FER-2013, with ResNet achieving the highest accuracy (90.14%) in their tests.

Recent advancements in the classification process of emotion detection have led to diverse and innovative approaches. Allognon et al. [116] present a method for continuous emotion detection using a CNN for feature extraction, a Convolutional Autoencoder for representation learning, and a Support Vector Regression (SVR) performed before the classification, achieving Concordance Correlation Coefficient (CCC) values of 51.6% for Valence and 26.4% for Arousal on the RECOLA dataset. Tu et al. [120] employ the Emotion6 Video Dataset and Ekman-6 Dataset, with distinct characteristics impacting model performance. The Emotion6 Video Dataset is synthetic, created from static images with clearly labeled emotions, which led to a high accuracy of 99.7%. Conversely, the Ekman-6 Dataset features more naturalistic and varied emotional expressions, resulting in a lower accuracy of 49.3%. Both datasets were divided into 70% for training, 15% for validation, and 15% for testing. Adding to these advancements, Sassi et al. [62] employ an approach by combining feature extraction using a CNN with classification via a Random Forest, leading to a 71.86% accuracy on the FER-2013 dataset. Muthamilselvan et al. [67] employ a CNN-based method augmented with binary whale optimization to detect emotions, demonstrating accuracies of 64.98%, 98.35%, 96.6%, and 99.42% across the SFEW, CK+, JAFFE, and FERG datasets, respectively. The datasets were split into 80% for training, 10% for validation, and 10% for testing, using the Adam optimizer. Alrowais et al. [84] introduce a pipeline that employs a GoogleNet model for feature extraction and a quantum autoencoder for classification, achieving 98.91% accuracy on the CK+ dataset.

Further expanding the scope of classification methods, Souza et al. [88] introduce an object detection approach adapted to emotion detection from video data, employing the Grassmannian Learning Mutual Subspace Method (G-LMSM) combined with CNNs. In their methodology, the CNNs serve as the backbone for feature extraction, while the G-LMSM acts as the classifier. When tested on the AFEW dataset, this approach achieves an accuracy of 38.27%, showcasing its potential to improve emotion recognition in video data compared to similar classification methods.

Fine-tuning emerges as a solution to the challenge of adapting pre-trained models to specific datasets or tasks. This approach, which involves freezing feature extraction layers while retraining classification layers, is particularly useful when dealing with limited or specialized data, allowing for more accurate and tailored models. Several authors [85,94,100,107,110,127,129] adopt a fine-tuning approach, freezing feature extraction layers and retraining classification layers. Kousalya et al. [97] explore optimizers, achieving training and evaluation accuracies of 87.59% and 83.59% using Stochastic Gradient Descent (SGD) optimizer. Pabba et al. [99] create a dataset captured in virtual classrooms, achieving 76.90% accuracy with fine-tuning. Nguyen et al. [103] classify macro-expressions using 3D images and achieve accuracies of 69.01% and 78.70% using a multilayer CNN. Reddy et al. [115] compare fine-tuning with HoG + SVM, obtaining accuracies of 76.77% and 62.47% using MobileNet-V2. Kim et al. [68] propose additive pooling and progressive fine-tuning for FER, achieving accuracies of 93.20%, 50.70%, and 89.52% on CK+, JAFFE, and KDEF datasets, respectively. Yan et al. [69] propose three emotion detection methods, with DFSN-I achieving accuracies of 98.73% and 87.50% on Oulu-CASIA and CK+ datasets, respectively.

Gerard et al. [126] use an ensemble of 64 CNN classifiers for emotion recognition, attaining a peak accuracy of 41.3%. Bindu et al. [122] apply two CNNs for feature extraction from drivers’ macro-expressions, achieving high accuracy on JAFFE and MMI datasets.

Akshi [91] introduce MEmoR, an emotion recognition model that employed transfer learning and achieves 83.79% accuracy. In contrast, Nima et al. [130] and Pooya et al. [131] use a CNN and a Pyramidal Lateral Inhibition Neural Network (LIPNet) to recognize expressive expressions, reaching an accuracy of 85.1%.

Ninad et al. [133] propose a two-step facial emotion detection pipeline using CNNs and reach 96% accuracy. Simultaneously, lightweight solutions [93,134] target embedded systems and edge computing with improved results over state-of-the-art CNNs [104,119,124,132,136].

Several multi-modal approaches have also emerged [21,108,109,128], fusing various sources of information and utilizing architectures such as LSTM and transformers, where Tran et al. [21] achieved a precision of 63.6% in facial emotion expressions. Chaudhari et al. [87] introduce a multi-modal method. They integrate three pretrained self-supervised learning models with a transformer and attention-based fusion method, employing Fab-Net for extracting video modality features. This method focuses on facial features in videos. Tested on the RAVDESS Audio–Visual Dataset, it achieves an accuracy of 86.40%.

#### 3.3.2. Facial Micro-Expressions

A micro-expression is often defined as an unintentional pattern of the human body that is significant enough to be seen yet too fleeting to indicate an emotion. Facial micro-expressions might be missed during casual observation or can be quite challenging to see in some cases [11].

Yanju et al. [41], propose an alternative to using CNN for micro-expression detection by using ViT for feature extraction. ViT has achieved better results than traditional CNN methods in image classification problems in recent years. It divides an image into a string of consecutive, non-overlapping image blocks, and then uses multi-headed self-attention in the transformer to learn features between the blocks in the image phase. This processing comes at a high processing cost, so the authors propose a method for micro-expression detection that consists of using Apple’s proposed MobileViT model, which combines the lightweight CNN MobileNetV2 with ViT to take advantage of the characteristics of both. To mitigate the problem of missing images with micro-expressions, the transfer learning technique is used to achieve high precision by combining a small dataset with micro-expressions from the following datasets: CASME II, SAMM, and SMIC. Thus, it is intended to train the MobileNet network with a custom dataset of macro-expressions and remove the weights of its training with higher precision. These weights are later used to train the MobileViT network for micro-expression detection. The custom dataset with macro-expressions was combined from the following datasets: CK+, Oulu-CASIA NIR&VIS facial expressions, and MUG facial expressions. Finally, a SVM was used for the classification phase because it has the greatest capacity to detect as many differences as possible. The result is a high accuracy of 84.27% with a processing time of 35.4 ms for a single image.

To reduce the problem of a low number of images for training micro-expressions models, Jelena et al [121] contribute to the growth of micro-expressions datasets. They analyze and catalog long videos with hidden sadness, and propose an algorithm for sadness detection with micro-expressions. The images used to enlarge the data set are taken in the laboratory, and subjects are asked to watch a video. Subjects are not informed of the purpose of the experience, allowing for the capture of spontaneous emotions. The intention is to catalog the captured images into three classes: Sad (obvious sadness), Neutral, and Blocked (hidden sadness). For feature extraction, critical points (landmarks) are collected from the whole face and eyes only. For classification, the SVM, Random Forest (RF), and CNN (AlexNet and VGG-16) methods are compared. The models trained using only the features extracted from the eye region provide the most accurate results. They achieve an accuracy of 99.08% for RF, 67.23% for SVM, and an average of 87.87% for CNN using a controlled environment.

Sun et al. [96] present an emotion detection strategy using a DL model in conjunction with an LSTM [105]. Due to the low performance of traditional approaches, the authors present a different method that encapsulates the network and gives better results in an indoor environment. Tran et al. [101] propose a new dataset for micro-expression detection, SMIC-E-Long an extension of the SMIC-E database, and test this dataset using a CNN for apex frame detection and then an LSTM and a Histogram of Image Gradient Orientation for Three Orthogonal Planes (HIGO-TOP) for classification. This method achieves an accuracy of 97% in a controlled environment. Other authors also attempt to integrate macro- and micro-expression recognition techniques, such as Liong et al. [106], who propose a CNN (SOFTNet) of three shallow optical streams for predicting an emotion that yields relevant results when tested in a workshop in 2020.

Madhumita et al. [78] enhance emotion detection by employing data augmentation, pre-processing, and face detection using OpenCV, and then creating a custom CNN. This CNN is trained and evaluated across a combination of CASME, CASME II, and CASME + 2 datasets, achieving accuracies of 74.25%, 75.57%, and 78.02% respectively. Leong et al. [80] used as well CNNs, and introduce an innovative approach in their study for emotion detection in videos. They utilize a lightweight 3DCNN backbone based on P3D ResNet integrated with an LSTM layer. Their method achieves accuracies of 71.1% and 61.9% on the CASME II and SAMM datasets, respectively.

#### 3.3.3. Gestures Expressions

In this review, gestures refer to the non-verbal cues related only with body movements that people use to communicate their emotions captured from a frame sequence. These gestures might be important for emotion detection, as they often convey more information about a person’s feelings than spoken words. In recent years, advancements in computer vision and machine learning have enabled researchers to develop algorithms that can accurately recognize and interpret body gestures in real time, making it possible to automate the process of emotion detection [12,141]. This section addresses DL methods to detect emotion using body gestures.

Romeo et al. [12] explore four state-of-the-art classification methods (3DCNN 3DResNet, CNN + LSTM VGG DAN+) for body gesture recognition, and all architectures of these methods have the temporal perception that brings advantages to the gesture detection scope. The MHHRI dataset is used and modified to include two-second clips from only one person; these are the input received by the methods for classification. To process these clips and to ensure that only one person appears in the clip, the authors use a standard R-CNN. The most successful method is the 3DCNN, with an accuracy of 62%.

Ilyas et al. [111] suggest a method that summarizes the characteristics of face and body gestures to detect emotions. It uses CNNs for feature extraction and classification of individual emotions and then applies an LSTM to use sequential information. For fusion, a shallow depth CNN is used to classify the final emotion. Using this approach, the authors are able to achieve 94.4% accuracy.

Hiranmayi et al. [22] present a multimodal dataset with data from human facial, body gestures, voice, and physiological signal features. The dataset consists of audio and video sequences of individuals expressing three different intensities of expression of 23 different emotions. People are asked to express emotions (posed emotions) indoors. The author also describes some model variations for training and evaluation using the proposed dataset. In this systematic literature review, only what is more accurate and relevant is mentioned, which is the model trained to recognize emotions using facial, body gesture, voice, and physiological signal data with 83.18% accuracy.

Prakash et al. [63] propose an approach for emotion detection in children with autism using videos. Their model combines a pre-trained ViT for gestures detection and a Resnet-34 model for facial macro-expression recognition. The study analyzes gestures and macro-expressions on YouTube videos and AVA dataset, and an adapted FER-2013 dataset, achieving accuracies of 72.32% and 95.1%.

#### 3.3.4. Static Poses Expressions

Static poses are positions and postures of a person’s body that are captured in a still image. Emotion detection can use these poses to determine a person’s emotional state by examining their pose body language and facial expressions. This is done using computer vision and machine learning techniques, which have been trained on a vast amount of data-associating images with emotional labels [142]. This section addresses emotion detection using the static pose expressions method.

Ronak et al. [13] present a new dataset for emotion recognition in context. The dataset catalogs with 23571 images from the Internet and the COCO dataset, which annotates a total of 34320 emotions of people cataloged into 26 different emotions. The dataset contains multiple images of people in different contexts, activities, and emotional states. The author proposes an architecture divided into three modules to test the dataset quality: fine-tuned CNN with an AlexNet for extracting features from static poses, CNN for feature extraction from the image context, and applied a fusion network of features for emotion classification. The test results are not satisfactory, with an accuracy of 27.38%. The author describes in more detail the relevance of using contextual information to detect emotions. Compared to other studies that use temporal context to improve classification performance, as in the aforementioned studies on emotion recognition using body gestures in Section 3.3.3, this study does not yield a relevant performance.

## 4. Discussion

The field of emotion recognition using computer vision and DL methods has experienced significant growth in recent years. Previous studies have analyzed several techniques for recognizing emotions in images and videos, including traditional computer vision methods such as R-CNN as well as newer approaches such as vision transformers [48]. These studies have shown that DL methods, especially those based on CNNs and transformers, have outperformed traditional computer vision techniques in terms of accuracy and robustness.

One of the major challenges in emotion recognition is the limited availability of labeled datasets. To address this issue, several large datasets have been created, such as the FER-2013 dataset, which consists of over 35,000 images labeled with seven basic emotions. These datasets have been instrumental in advancing the state-of-the-art in emotion recognition by allowing researchers to train and test their algorithms on a large and diverse dataset.

Overall, the results of previous studies have shown that computer vision and DL methods are very promising for the field of emotion recognition. However, there is still much room for improvement, and further research is needed to address challenges such as handling expressions in different cultural contexts, dealing with occlusions and incomplete faces, and improving the interpretability of learned representations. These are important avenues for future research in emotion recognition using computer vision and DL methods.

In this section, we analyze the results of the studies included in the previous section on emotion recognition using computer vision and DL methods, divided into different expression types. The initial research questions gathered insights.

### 4.1. RQ1 What Type of Emotion Expressions Are Addressed in Literature?

In general, the analysis of the studies reviewed in this SLR, addressed in Table 9, shows that the focus of research is on the use of macro-expressions for FER with more than 88.3% of the studies reviewed. The reason for this is that they are considered universal, easily recognizable, and reliable indicators of emotion. They are thought to have evolved as a means of communicating emotions across cultures and are based on biologically determined facial muscles. These expressions are captured in still images or video frames and are the focus of emotion recognition algorithms. Although they are easy to recognize, their applicability in a real-world context is still challenging. The second expression type with the highest number of studies is micro-expressions at 11.7%, although there are few studies that are important for emotion recognition because they provide additional, nuanced information about a person’s emotional state. Unlike macro facial expressions, which are typically associated with more intense emotions, micro facial expressions may occur in response to a broader range of emotional states and reveal subliminal, unconscious emotions. Incorporating micro-facial expressions into emotion recognition algorithms may help increase their accuracy and robustness.

### 4.2. RQ2 What Are the Deep Learning Methods Utilized?

In general, the methods used in the studies reviewed were mainly based on CNNs at 84.4%, with transfer learning being the most commonly used technique. Some studies used existing state-of-the-art CNN that were pre-trained for emotion recognition, while others proposed their own CNN architectures or made strategic changes to improve accuracy. CNNs are particularly well suited for this task because they can automatically learn and extract features from images, making them suitable for analyzing complex patterns in facial expressions. The second most frequently used method was Faster R-CNN, which accounted for 6.5% of the trials. This method has been used primarily for face identification in image processing and is known for its high precision, albeit at the cost of demanding computational resources. Since it is a new type of CNN and few studies are available so far, Faster R-CNN holds great potential for further investigation. The third most commonly used method was ViT at 10.3%. Unlike older computer vision techniques such as R-CNN, vision transformers do not require predefined regions of interest and can use the global context in the image to make a prediction. This can lead to improved accuracy and robustness in emotion recognition compared to other approaches. ViTs are a really interesting area of research due to their potential and novel approaches. Other neural networks that were used by the reviewed studies corresponding to 6.5% of the studies reviewed are alternatives to CNNs and their variants.

### 4.3. RQ3 Which Datasets Are Employed by Relevant Works?

The dataset is a crucial factor in the training process of DL models. The accuracy and performance of the model are highly influenced by the quality and size of the dataset. The dataset must be representative of the real-world data the model will face and contain enough samples to effectively train the model. It should also be balanced and diverse, covering a range of emotions, facial expressions, and poses to prevent model bias and improve performance. Table 10 shows how many datasets were created and proposed in the studies analyzed. The lack of data for training or evaluating a method is the main reason for the author or authors’ decision to create a dataset, which was the case in 15.6% of the studies analyzed. Although the quality of datasets has improved over time, it is still a major challenge to achieve high accuracy when testing in an uncontrolled environment. The vast majority of studies reviewed (84.4%) used existing datasets, generally due to the complexity of capturing and cataloging images. Below is a list of the most commonly used datasets, grouped by expression type.

- Facial macro-expressions—FER-2013, CK+, JAFFE, e RAF-DB
- Facial micro-expressions—CASME II, SMIC, CK+
- Body gesture expressions—MHHRI
- Static pose expressions—EMOTIC

### 4.4. RQ4 Which Performance Improvement Techniques Can Be Employed in This Context?

**Figure 4:**
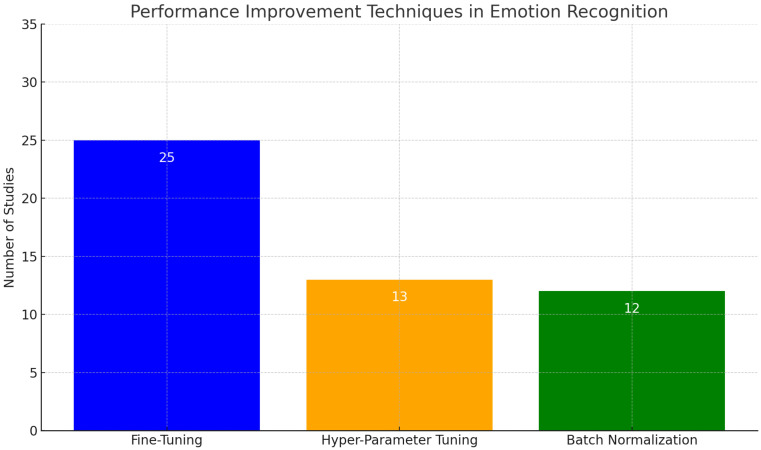
Number of studies employing performance improvement techniques. The graph compares the number of studies that mentioned the use of fine-tuning, hyper-parameter tuning, and batch normalization, out of a total of 77 studies fully analyzed.

Recent advances in the field of emotion recognition using DL methods have focused on improving the accuracy and robustness of the models. A key area of focus has been on fine-tuning pre-trained models, hyper-parameter tuning, and batch normalization as effective performance improvement techniques. Among the reviewed studies, as shown in Figure 4, fine-tuning was the most mentioned technique for performance improvement.

It is important to note that these numbers may not reflect the actual values, as they are based on our interpretation of the articles and different levels of detail provided in the papers. Additionally, innovative DL architectures such as ViT have garnered increased attention in recent research for their superior performance compared to traditional CNNs.

Another area of focus is the use of transfer learning techniques, where pre-trained models are adapted to specific tasks to reduce training time and improve performance. Moreover, researchers have explored multi-modal approaches, incorporating facial, body, and/or audio data for a more comprehensive understanding of emotions.

In addition, researchers have worked to develop models that can handle “in the wild” scenarios, where data are collected and tested in real-world environments and can exhibit a high degree of variability. Overall, these advances help advance the field of emotion recognition using DL methods and improve the accuracy and robustness of the models. Based on the reviewed studies presented in Table 6 and Table 11, the most commonly used method, CNN, was adapted with transfer-learning. The research field in this area still has much room for improvement, namely the use of Faster R-CNNs and ViTs.

### 4.5. Lessons Learned

In Section 3, we conducted a detailed analysis of the literature on emotion recognition using DL techniques. Our research yielded valuable insights and lessons learned.

Emotion recognition using DL is a popular research area that has yielded several approaches for achieving accurate results. Among the most commonly used methods are CNNs. Custom CNN architectures or modifications to existing models have also been proposed for better results. Additionally, the Faster R-CNN method is mainly used for face recognition in image processing. In the context of emotion detection, studies have shown that body-related expressions generally yield worse results compared to facial-related expressions.

Preprocessing techniques play a crucial role in emotion recognition. Common techniques include face detection and alignment, color normalization, cropping and resizing, data augmentation, and image noise reduction. By applying these techniques, researchers can ensure that the input data is of high quality and suitable for use in emotion recognition models.

Recently, ViT has emerged as a promising DL technique for emotion recognition. It is particularly well-suited for image and video analysis, given its ability to process large amounts of data while maintaining high accuracy. ViTs achieve this through their self-observation mechanism, which enables the analysis of relationships between elements of the input data. They can effectively model spatial relationships, making them a preferred choice for emotion recognition. Although vision transformers have shown exceptional results in the context of NLP, there is still much room for development in their application to emotion recognition tasks. Researchers can continue to explore the potential of ViTs for emotion recognition tasks and identify ways to enhance their performance.

Despite the promise of DL techniques for emotion recognition, several challenges remain. The most significant challenges include the lack of large and diverse training datasets, which GANs can address by synthesizing realistic data to mitigate dataset scarcity. Another challenge is the lack of annotated data that limits the improvement of accuracy of emotion recognition models, and the need for high-performance hardware for real-time, low-latency processing. One of the major challenges in the field of emotion recognition is the lack of public datasets containing spontaneous macro- and micro-expressions. Addressing this issue with the help of GANs could significantly contribute to the development of accurate emotion recognition models that can be applied in real-world contexts.

Moreover, exploring emotion recognition using whole-body information could make a significant contribution to recognizing customer feedback in a real-world context. This approach would involve analyzing not only facial expressions but also body language and vocal cues to gain a more comprehensive understanding of the customer’s emotional state. This could help businesses improve their customer service and overall customer experience.

Few studies focus on detecting emotions in specific populations such as individuals with autism, elderly people, or those who display subtle or non-visual emotional cues. This scarcity is due to the complex challenge inherent in discerning emotions within these groups, where traditional computer vision techniques might not be sufficient for accurate emotion detection. Moreover, there is not sufficient research in this area to draw definitive conclusions about the differences between DL methods and traditional techniques. These challenging groups often exhibit unique characteristics, such as atypical facial expressions, reduced or exaggerated emotional responses, and non-visual cues, which complicate the detection of emotions [143,144].

## 5. Conclusions and Future Work

This paper presented an SLR for emotion recognition using computer vision and DL methods. It is intended that this review gives an overview of the state of the art of the mentioned topic and helps researchers to understand the trends in the field of emotion recognition in facial and body poses. First, we analyzed the main databases of scientific documents, selecting 77 studies for review. Second, we summarized the studies and extracted the main features such as the type of emotion recognized in the study and the main methods and datasets used. Third, these studies were compared and analyzed to describe the most important information. In recent years, interest in emotion recognition has increased, and it is used in areas such as police interrogation, traffic safety, clinical analysis, and many others. The field of emotion recognition using computer vision and DL methods has made great strides in recent years. CNNs are widely used in this field because they are very accurate and robust compared to traditional computer vision techniques. However, the scarcity of annotated data and hardware requirements remains a major challenge in this field. Newer techniques, such as vision transformers, promise even greater advances in the future. Further research and advances are needed to further improve the accuracy and applicability of real-world emotion recognition models. The use of macro-expressions is currently the most researched area of emotion recognition, which despite its high scientific content, still has some challenges to overcome, such as recognition in an uncontrolled environment due to the number of variations that occur, brightness, and facial rotation. On the other hand, micro-expressions are now gaining greater focus due to the emergence of new technologies, namely transformers and newer region methods (Faster R-CNNs). Currently, there are issues related to datasets that do not contain enough data on spontaneous expressions to train a model. Finally, hand and body expressions are not as much the focus of researchers, probably because most human communication is done through the face and not the body.

## Figures and Tables

**Table 1:** Comparison with previous reviews. (✓: Discussed, ✗: Not Discussed).

Reference	Research Area (FER)	Research Area (PER)	Region Methods	Vision Transformer Methods	Datasets
[9]	✓	✗	✗	✗	✓
[26]	✓	✗	✗	✗	✓
[27]	✓	✗	✗	✗	✓
[28]	✓	✗	✗	✗	✗
[29]	✓	✗	✗	✗	✓
[30]	✓	✓	✗	✗	✗
[31]	✓	✗	✓	✗	✗
[14]	✓	✗	✗	✗	✓
Current Review	✓	✓	✓	✓	✓

**Table 2:** Quality Assessment (QA) questions.

#QA	Quality Questions	Yes	Partially	No
QA1	Are the objectives of the study clearly identified?	32	45	8
QA2	Are the limitations of the study clearly specified?	15	19	51
QA3	Are the detected emotions clearly specified?	48	24	13
QA4	Are the technologies used clearly specified?	23	54	8
QA5	Are the emotion detection targets clearly specified?	10	4	71
QA6	Are the used dataset clearly specified?	76	4	5
QA7	Are the findings and results stated and discussed?	64	16	5
QA8	Is the research methodology reproducible?	14	22	49
QA9	Has a comparative analysis been performed?	45	23	17

**Table 3:** Quality Assessment (QA) answers.

#	Rank	Category	Studies
1	9 ≥ score > 8	Very high	1
2	8 ≥ score > 6.5	High	16
3	6.5 ≥ score > 4.5	Medium	31
4	4.5 ≥ score > 2.5	Low	29
5	2.5 ≥ score > 0	Very Low	8

**Table 4:** Mostly used emotion recognition datasets.

Expression	Database	Posed/ In the Wild	Images/Videos	Type	Subjects	Expressions	References
Facial Macro	FER-2013 [60]	in the wild	35,000 images	gray	-	anger, disgust, fear, happiness, sadness, surprise, neutral	[61,62,63]
	CK+ [64]	posed	593 images	mostly gray	123	neutral, sadness, surprise, happiness, fear, anger, contempt, disgust	[61,65,66,67,68,69]
	JAFFE [70]	posed	213 images	gray	10	neutral, sadness, surprise, happiness, fear, anger, disgust	[61,67,68,71]
	RAF-DB [72]	in the wild	29,000 images	color	300+	anger, disgust, fear, happiness, sadness, surprise, neutral	[72]
	AffectNET [73]	in the wild	950,000	color	-	neutral, happiness, sadness, surprise, fear, disgust, anger, and contempt	[45,74]
	Aff-Wild2 [75]	in the wild	2,800,000	color	458	neutral, happiness, sadness, surprise, fear, disgust, anger + valence–arousal + action units 1, 2, 4, 6, 12, 15, 20, 25	[76]
Facial Micro	CASME [77]	posed	195 images	color	19	amusement, sadness, disgust, surprise, contempt, fear, repression, tense	[78]
	CASME II [79]	posed	247 images	color	26	happiness, disgust, surprise, repression, and others	[40,41,78,80]
Gestures	MHHRI [81]	posed	48 videos	color	18	self-/acquaintance-assessed personality, self-reported engagement	[12]
Static Poses	EMOTIC [82]	in the wild	18,313 images	color	18	anger, fatigue, fear, happiness, sadness, pain, confidence, and others	[13]

**Table 5:** Abbreviations for the summary.

Dimension	Category
Scope	S1: Macro-Facial Expressions
	S2: Micro facial expressions
	S3: Static pose expressions
	S4: Body gesture expressions
Test Environment	TE1: In the wild
	TE2: controlled
Methods	M1: CNN
	M2: Faster R-CNN
	M3: Vision transformers
	M4: Other NNs
Datasets	D1: Created
	D2: Already exists

**Table 6:** Summary based on the proposed taxonomy and the abbreviations in Table 5.

Reference	Scope	Test Environment	Method	Dataset	Year
[83]	S1	TE2	M1	D1	2022
[80]	S2	TE2	M4	D2	2022
[84]	S1	TE2	M4	D2	2023
[85]	S1	TE2	M1	D2	2023
[63]	S1 and S4	TE2	M1 and M3	D1	2023
[86]	S1	TE2	M1	D2	2023
[87]	S1	TE2	M3	D2	2023
[67]	S1	TE2	M1	D2	2023
[74]	S1	TE2	M1	D2	2023
[88]	S1	TE2	M4	D2	2023
[40]	S1 and S2	TE2	M3	D2	2023
[89]	S1	TE2	M3	D2	2023
[90]	S1	TE2	M1	D2	2023
[62]	S1	TE2	M1	D2	2023
[66]	S1	TE2	M1	D2	2022
[91]	S1	TE2	M1	D2	2022
[41]	S2	TE2	M3	D2	2022
[92]	S1	TE2	M3	D2	2022
[93]	S1	TE2	M1	D2	2022
[94]	S1	TE2	M1	D2	2022
[95]	S1	TE2	M1	D1	2022
[61]	S1	TE2	M1 and M2	D2	2022
[96]	S2	TE2	M1	D2	2022
[21]	S1	TE2	M3	D2	2022
[97]	S1	TE2	M1	D2	2022
[98]	S1	TE2	M1	D2	2022
[99]	S1	TE1	M1	D1	2022
[100]	S1	TE2	M1	D2	2021
[101]	S2	TE2	M1	D1	2022
[102]	S1	TE2	M1	D2	2021
[103]	S1	TE2	M1	D2	2021
[104]	S1	TE2	M4	D2	2021
[105]	S2	TE2	M1	D2	2021
[106]	S1 and S2	TE2	M1	D2	2021
[45]	S1	TE1	M1	D2	2021
[107]	S1	TE1	M1	D2	2021
[76]	S1	TE1	M1	D2	2021
[108]	S1	TE1	M1 and M2	D2	2021
[52]	S1	TE2	M1	D2	2021
[12]	S4	TE2	M2 and M1	D2	2021
[109]	S1	TE1	M2 and M1	D2	2021
[110]	S1	TE1	M1	D1	2021
[111]	S1 and S4	TE1	M1	D2	2021
[112]	S1	TE1	M1	D2	2021
[113]	S1	TE2	M3	D2	2021
[114]	S1	TE2	M1	D2	2020
[115]	S1	TE1	M1	D2	2020
[116]	S1	TE2	M1	D2	2020
[117]	S1	TE1	M1	D2	2020
[118]	S1	TE1	M1	D2	2020
[119]	S1	TE1	M1	D2	2020
[120]	S1	TE2	M1	D2	2020
[71]	S1	TE2	M4	D2	2020
[121]	S2	TE1	M1	D1	2019
[122]	S1	TE2	M1	D2	2018
[123]	S1	TE2	M1	D1	2018
[124]	S1	TE1	M1	D2	2018
[69]	S1	TE2	M1	D2	2018
[125]	S1	TE2	M1	D2	2018
[126]	S1	TE2	M1	D2	2018
[127]	S1	TE2	M1	D2	2018
[65]	S1	TE2	M1	D2	2018
[78]	S2	TE2	M1	D2	2017
[72]	S1	TE2	M1	D1	2017
[128]	S1	TE2	M1	D2	2017
[129]	S1	TE2	M1	D2	2017
[130]	S1	TE2	M1	D2	2016
[22]	S1 and S4	TE2	M1	D1	2016
[131]	S1	TE2	M1	D2	2015
[132]	S1	TE1	M1	D2	2021
[13]	S3	TE2	M1	D1	2020
[133]	S1	TE2	M1	D2	2020
[134]	S1	TE1	M1	D2	2021
[135]	S1	TE1	M1	D2	2021
[136]	S1	TE1	M2 and M1	D1	2022
[137]	S1	TE1	M1	D2	2022
[68]	S1	TE1	M1	D2	2018

**Table 7:** Abreviations for the created datasets summary.

Dimension	Category
Emotion type	ET1: Posed
	ET2: Spontaneous
Capture environment	CE1: Outdoor
	CE2: Indoor

**Table 8:** Summary of created datasets from reviewed research based on the proposed taxonomy and the abbreviations in Table 7.

Reference	Emotion Type	Capture Environment
[83]	ET1	CE1
[63]	ET2	CE2
[95]	ET2	CE1
[99]	ET2	CE2
[101]	ET2	CE2
[110]	ET2	CE1
[121]	ET2	CE2
[123]	ET1	CE2
[72]	ET2	CE1
[22]	ET1 and ET2	CE2
[13]	ET2	CE1
[136]	ET2	CE2

**Table 9:** Total of types of emotion problems.

Emotion Problems	Total	Percentage
Facial macro-expressions	68	88.3%
Facial micro-expressions	9	11.7%
Static pose expressions	1	1.3%
Body gesture expressions	4	5.2%

**Table 10:** Origin of datasets.

Origin	Total	Percentage
Created	12	15.6%
Already exists	65	84.4%

**Table 11:** Total deep learning methods utilized.

Deep Learning Methods	Total	Percentage
CNN	65	84.4%
Faster R-CNN	5	6.5%
Vision transformers	8	10.3%
Other NNs	5	6.5%

## Abbreviations

The following abbreviations are used in this manuscript:

AI	Artificial Intelligence
DL	Deep Learning
CNN	Convolutional Neural Network
ER	Emotion Recognition
FER	Facial Emotion Recognition
PER	Pose Emotion Recognition
HoG	Histogram Oriented Gradient
SLR	Systematic Literature Review
SVM	Support Vector Machine
LSTM	Long Short Term Memory
R-CNN	Region-based Convolutional Neural Networks
ViT	Vision Transformers
NLP	Natural Language Processing
RoI	Region of Interest
AU	Action Units
EoT	Eyes of Things
GAN	Generative Adversarial Networks
YOLO	You Only Look Once
SSD	Single Shot MultiBox Detector
CCC	Concordance Correlation Coefficient
ANN	Artificial Neural Network
DeepFEVER	Deep Facial Expression Vector ExtractoR
FgbCNN	Fine-Grained Bilinear CNN
FT-CSAT	Fine-Tuned Channel-Spatial Attention Transformer
SPT	Shifted Patch Tokenization
LSA	Locality Self-Attention
FPS	Frames Per Second
G-LMSM	Grassmannian Learning Mutual Subspace Method
SVR	Support Vector Regression
SGD	Stochastic Gradient Descent
WOA	Whale optimization algorithm
DLP	Deep Locality-Preserving
LDA	Linear Discriminant Analysis
dacssGAN	Domain Adaptation Conditional Semi-Supervised Generative Adversarial Network
MAL	Memo Affinity Loss
MAFT	Mask Attention Fine Tuning
PRISMA	Preferred Reporting Items for Systematic Reviews and Meta-Analyses
NLU	Natural Language Understanding
HCI	Human–Computer Interaction
EmotiW	Emotion Recognition in the Wild
RAF-DB	Relaxed Affective Faces Database
